# Pan‐Cancer Single‐Cell Transcriptomic Analysis Reveals Divergent Expression of Embryonic Proangiogenesis Gene Modules in Tumorigenesis

**DOI:** 10.1002/cam4.70373

**Published:** 2024-11-11

**Authors:** Zeshuai Wang, Yiyi Su, Lisha Zhao, Wei Liu, Jiaqi Zhang, Wei Yang, Hanjie Li, Mingqian Feng, Hao Wang, Zhuo Song

**Affiliations:** ^1^ College of Life Science and Technology Huazhong Agricultural University Wuhan China; ^2^ Maternal‐Fetal Medicine Institute, Department of Obstetrics and Gynecology, Shenzhen Baoan Women’s and Children’s Hospital Shenzhen University Shenzhen China; ^3^ Guangdong Provincial Key Laboratory of Medical Molecular Diagnostics Guangdong Medical University Dongguan China; ^4^ Key Laboratory of Quantitative Synthetic Biology Shenzhen Institute of Synthetic Biology, Shenzhen Institute of Advanced Technology, Chinese Academy of Sciences Shenzhen China

**Keywords:** angiogenesis, angiogenic macrophages, embryonic development, neovascularization, single‐cell transcriptome, tumor microenvironment

## Abstract

**Background:**

Angiogenesis is indispensable for the sustained survival and progression of both embryonic development and tumorigenesis. This intricate process is tightly regulated by a multitude of pro‐angiogenic genes. The presence of gene modules facilitating angiogenesis has been substantiated in both embryonic development and the context of tumor proliferation. However, it remains unresolved whether the pro‐angiogenic gene modules expressed during embryonic development also exist in tumors.

**Methods:**

This study performed a pan‐cancer single‐cell RNA sequencing (scRNA‐seq) analysis on samples from 332 patients across seven cancer types: thyroid carcinoma, lung cancer, breast cancer, hepatocellular carcinoma, colorectal cancer, ovarian carcinoma, and prostate adenocarcinoma. Data processing was carried out using the Seurat R package, with rigorous quality control to filter high‐quality cells and mitigate batch effects across datasets. We used principal component analysis (PCA), shared nearest neighbor graph‐based clustering, and Uniform Manifold Approximation and Projection (UMAP) to visualize cell types and identify distinct cell clusters. Myeloid cell subpopulations were further analyzed for the expression of embryonic pro‐angiogenic gene modules (EPGM) and tumor pro‐angiogenic gene modules (TPGM).

**Results:**

The analysis identified nine major cell types within the tumor microenvironment, with myeloid cells consistently exhibiting elevated expression of both tumor pro‐angiogenic gene modules (TPGM) and EPGM across all tumor types. In particular, myeloid cells, including macrophages and monocytes, showed high EPGM expression, indicating an active role of embryonic pro‐angiogenesis pathways in tumors. A subset analysis revealed 20 distinct myeloid subtypes with varying EPGM and TPGM expression across different cancers. Treatment and disease stage influenced these gene expressions, with certain subtypes, such as HSPAhi/STAT1+ macrophages in breast cancer, displaying reduced pro‐angiogenic gene activity post‐treatment.

**Conclusion:**

This study provides evidence that tumors may exploit EPGM to enhance vascularization and support sustained growth, as evidenced by the elevated EPGM expression in tumor‐associated myeloid cells. The consistent presence of EPGM in TAMs across multiple cancer types suggests a conserved mechanism wherein tumors harness embryonic angiogenic pathways to facilitate their progression. Distinct EPGM expression patterns in specific myeloid cell subsets indicate potential therapeutic targets, particularly in cases where EPGM activation contributes to resistance against anti‐angiogenic therapies. These findings shed new light on the molecular mechanisms underlying tumor angiogenesis and highlight the prognostic relevance of EPGM expression in cancer, underscoring its potential as a biomarker for clinical applications.

## 1 Introduction

Angiogenesis is a fundamental process involved in embryonic development, organ growth, and tissue healing [1, 2, 3]. This process facilitates the delivery of oxygen and nutrients, which are crucial for these processes, and efficiently removes metabolic waste products from cells. Dysregulation of this process often leads to uncontrolled blood vessel growth, contributing to tumor development [4, 5]. Consequently, anti‐angiogenic therapies have emerged as promising strategies for treating cancer [6, 7, 8]. However, their effectiveness is limited, with some patients showing no response and others developing resistance [9, 10, 11]. Therefore, using the intricate mechanism of angiogenesis to hinder tumor onset and progression remains a considerable challenge. Thus, understanding the mechanisms that drive intricate angiogenic pathways in tumors is imperative for formulating precise interventions to impede tumor vascularization and halt the advancement of the disease [5, 12, 13, 14, 15].

The regulatory mechanisms governing tumor angiogenesis encompass a wide array of factors derived from different cell types, among which tumor‐associated macrophages (TAMs) play a critical role [15, 16, 17, 18, 19]. TAMs are concentrated primarily within the hypoxic regions of tumors, especially within the necrotic tissues. TAMs produce essential molecules such as vascular endothelial growth factor A (VEGFA), tumor necrosis factor (TNF), C‐X‐C motif chemokine ligand 8 (CXCL8), platelet‐derived growth factor, fibroblast growth factor 2 (FGF2), and matrix metallopeptidase (MMP). These molecules contribute significantly to the intricate process of tumor angiogenesis, promoting tumor expansion, invasion into nearby tissues, and potential dissemination to distant anatomical sites [20, 21, 22, 23].

Recent investigations have revealed significant similarities in the developmental processes of early embryos and tumorigenesis, including migration and invasion, gene expression, protein profiles, signaling pathways, cell differentiation, mechanisms of immune escape, and other relevant aspects [24, 25, 26, 27, 28]. Previous studies have shown similar behaviors of TAMs during tumorigenesis, similar to how embryos invade the uterine stroma after attaching to the endometrium [29]. Similarly, in our previous studies on the role of macrophages in embryonic angiogenesis, we identified a macrophage subtype, PraM. These cells exhibit a gene expression profile conducive to proangiogenesis across various tissue types, marked by increased expression of essential genes such as VEGFA, TNF, and CXCL8 [30, 31, 32]. While acknowledging that the vascularized microenvironment in tumors closely resembles that observed during embryonic development [27, 33, 34, 35, 36], it is noteworthy that the tightly controlled mechanisms regulating embryonic angiogenesis in tumors remain unclear. Tumors may harness an embryonic proangiogenic gene module (EPGM) to fuel their growth.

In this study, we conducted a comprehensive analysis of seven tumor types using a combination of published scRNA‐seq data. Through extensive data mining, we found significant expression of the EPGM within the tumor microenvironment, providing novel insights into the mechanisms underlying angiogenesis in associated cancers.

## 2 Materials and Methods

### 2.1 Analysis of Single‐Cell RNA‐Seq Data

We employed the Seurat R package (version 5.0.1), developed and maintained by the Satija Lab in the United States, to rigorously control and process single‐cell RNA sequencing (scRNA‐seq) data, thus ensuring the incorporation of only high‐quality cells for downstream analytical steps. Specifically, we assessed the distribution of key metrics—namely, gene expression coverage, the number of detected expressed genes (features) per cell, and mitochondrial gene expression percentage to total gene expression—across cells to identify an optimal valid data region [37].

To establish thresholds for gene expression coverage, we utilized quantiles of RNA counts (0.025 and 0.975) to delineate boundaries. Similarly, thresholds for the number of detected expressed genes per cell were determined using quantiles (0.025 and 0.975) of feature counts. Additionally, thresholds for mitochondrial gene expression percentage to total gene expression were established using quantiles (0.025 and 0.975). These thresholds facilitated the identification of outlier cells or cells of low quality within our dataset, ensuring that subsequent analyses were conducted on high‐quality data [38].

Subsequently, we normalized the count data to generate a normalized data matrix. In cases where specific datasets lacked count data, we directly utilized the TPM matrix downloaded from available sources. To mitigate batch effects, we employed Harmony (version: 1.2.0) for integration of all scRNA‐seq databases [39].

Principal component analysis (PCA) was performed as part of linear dimension reduction analysis using gene expression data [40]. Downstream analyses, including shared nearest neighbor graph‐based clustering, marker gene expression analysis, and visualization, were conducted using the Seurat R package. Identified marker genes for each cell classification were visualized using the “DotPlot,” “feature plot” functions, and “ggplot2” library. Clusters were delineated using the community identification algorithm implemented in the Seurat “FindNeighbors” and “FindClusters” functions [37].

The shared nearest neighbor graph was constructed using between 10 and 30 canonical correlation vectors, determined by dataset variability. The resolution parameter was adjusted to yield a sufficient number of clusters capturing most of the biological variability. Uniform Manifold Approximation and Projection (UMAP) analysis was executed using the “RunUMAP” function with default parameters. Finally, clusters were annotated using canonical cell‐type markers [41].

## 3 Results

### 3.1 ScRNA‐Seq Analysis Unveils Nine Major Cell Subtypes Across Seven Human Cancer Types

**FIGURE 1:**
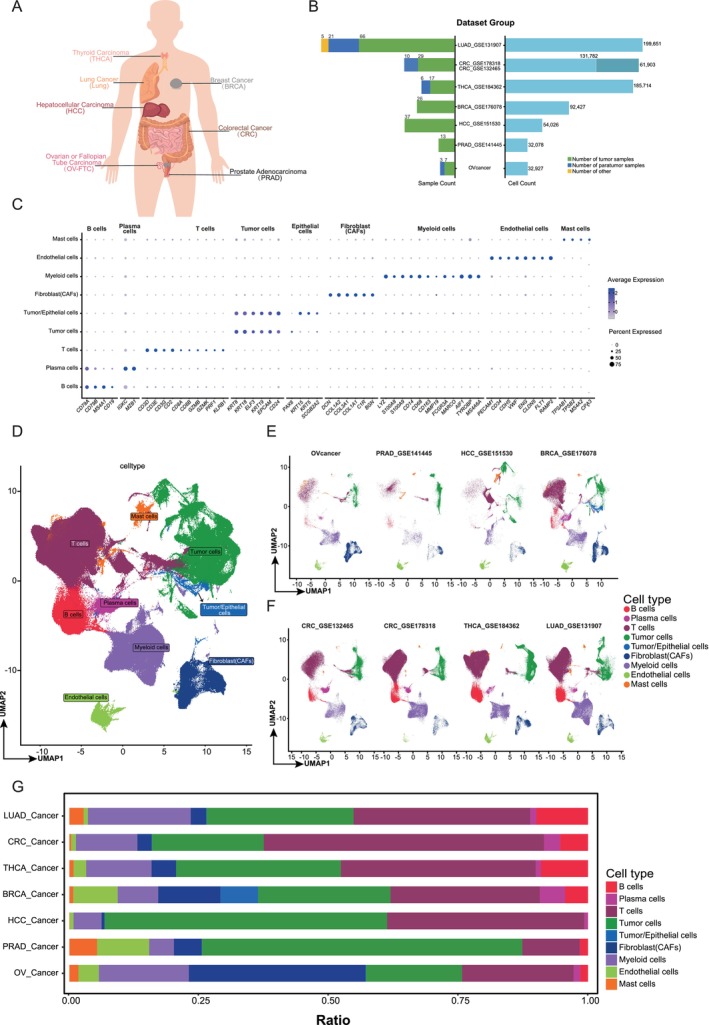
A pan‐cancer single‐cell transcriptome atlas. (A) Cancer types involved in the pan‐cancer study. (B) The number of sample counts and cell counts across cancer types. (C) Dot plot showing normalized expression of selected marker genes for the main lineages. The color represents the mean expression level, and the size indicates the proportions of cells expressing the genes. (D–F) Uniform manifold approximation and projection (UMAP) visualizations of all cancer types (D) and each cancer (E and F). (G) Barplots showing the main cell compositions of each cancer.

To analyze the heterogeneity of the tumor microenvironment, we integrated multiple single‐cell transcriptomic datasets obtained from public databases for thyroid carcinoma (THCA), lung cancer (LUAD), breast cancer (BRCA), hepatocellular carcinoma (HCC), colorectal cancer (CRC), ovarian/fallopian tube carcinoma (OV‐FTC), and prostate adenocarcinoma (PRAD) (Figure 1A; Table S1). This compilation allowed us to create a pan‐cancer map comprising 332 samples and 804,406 cells (Figure 1A,B). Using unsupervised graph‐based clustering, we characterized the subsets and removed batch effects from different datasets. Subsequently, we identified nine major common lineages based on canonical cell markers: mast cells, endothelial cells, myeloid cells, fibroblasts, tumor/endothelial cells, tumor cells, T cells, plasma cells, and B cells (Figure 1C). Cell types with annotations were visualized using a UMAP, and the results showed notable heterogeneity across tumor types (Figure 1D,E; Section 2). To clarify the cell‐type distribution across tumors, we employed stacked bar graphs to illustrate the cellular composition of seven tumor samples, emphasizing distinct cell participation patterns across tumor environments. Notably, myeloid cells exhibited consistent representation across many tumor types, suggesting a pervasive and pivotal role in tumor development (Figure 1F).

### 3.2 Tumor Myeloid Cells Exhibit Heightened Expression of the Embryonic Proangiogenesis Gene Module

**FIGURE 2:**
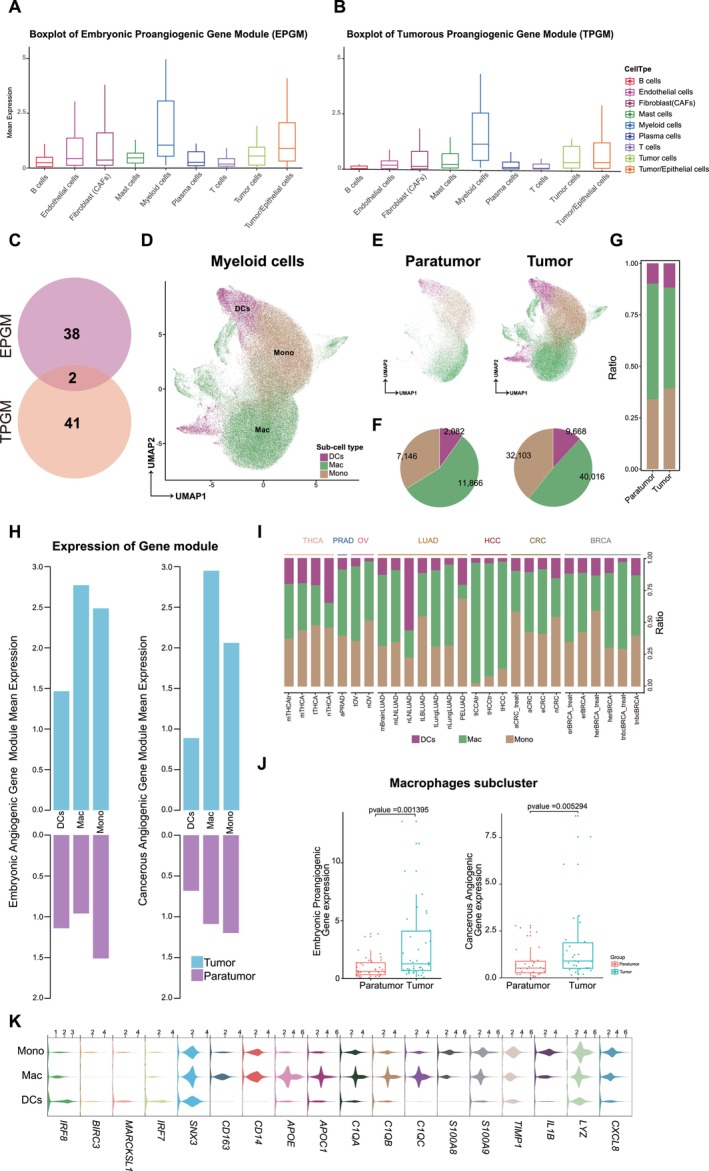
Analysis of myeloid cell lineages and proangiogenic gene modules in tumors. (A and B) Boxplots showing the mean expression of embryonic proangiogenic (A) and tumorous proangiogenic (B) gene modules in tumors. (C) The number of genes present in the EPGM and tumorous proangiogenic gene module (TPGM), as well as the shared genes between the two modules. (D) UMAP visualizations illustrating myeloid cell lineages across all cancer types. (E) 2D‐projection as in D, in which para tumor and tumor regions are depicted, respectively. (F) Pie chart displays the counts of various myeloid cells in the para tumor and tumor regions. (G) Barplots showing the myeloid cell compositions in both the para tumor and tumor regions. (H) Violin plot showing the expression distribution of the selected marker genes of monocytes (mono), macrophage (mac), and dendritic cells (DCs). (I) Bar chart showing the mean expression of EPGM and TPGM in mono, mac, and DCs from both paratumor and tumor regions. (J) Barplots showing the myeloid cell compositions for each cancer type. (K) Boxplot showing the expression levels of EPGM and TPGM in macrophages subcluster within both paratumor and tumor regions. The p‐value of the Wilcoxon test between different groups is indicated.

Previous studies have underscored the importance of the tumor‐associated proangiogenic gene module (TPGM) [18, 19, 42, 43, 44, 45]. However, the status of EPGM in the tumor environment remains unexplored. To investigate the role of EPGM in tumorigenesis, we evaluated its expression across all cell subtypes in a pan‐tumor context. We found that TPGM was highly expressed in the tumor environment, particularly in myeloid cells (Figure 2A). We also observed high expression of EPGM in myeloid cells (Figure 2B). Using a Venn diagram, we compared the two gene modules (TPGM and EPGM) and found that only two genes overlapped, indicating a limited similarity (Figure 2C; Tables S1 and S2; Section 2). These results indicate that myeloid‐derived cells concurrently activate both gene modules in the tumor environment.

Myeloid cells were divided into three primary subpopulations: monocytes (mono), dendritic cells (DC), and macrophages (Macs) (Figure 2D). When comparing tumor sites to adjacent normal cells, monocytes and macrophages were dominant in both cell counts and proportions (Figure 2E–G,I). A detailed analysis showed heightened TPGM and EPGM expression in tumors compared to normal tissues (Figure 2H,K). Further quantitative analysis revealed significant overexpression of both TPGM and EPGM in macrophages in adjacent normal tissues and tumors. (Figure 2J). Overall, these findings suggest that activation of EPGM in myeloid‐derived cells plays a pivotal role in driving angiogenesis in the tumor microenvironment.

### 3.3 Pan‐Cancer Analysis of the Diversity of TPGM and EPGM Expression in Myeloid Cell Subsets

**FIGURE 3:**
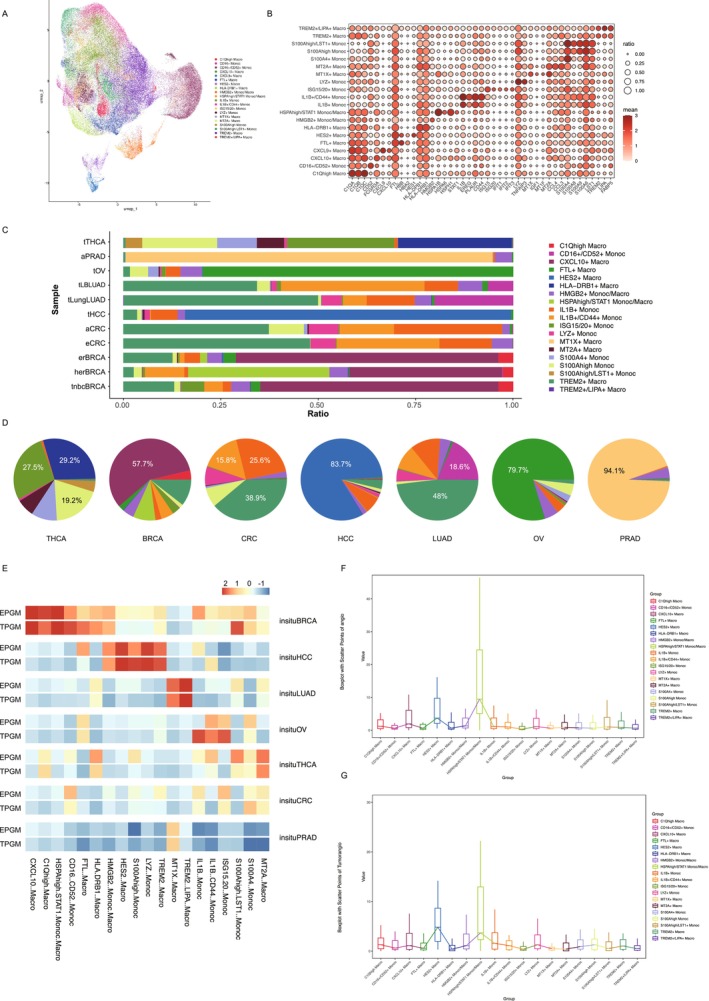
Single‐cell analysis of macrophage subtypes in a pan‐cancer transcriptome atlas. (A) UMAP visualizations of macrophage subtypes. (B) Dotplot showing normalized expression of selected marker genes for the macrophage subtypes. The color represents the mean expression level, and the size indicates the proportions of cells expressing the genes. (C) Barplots showing the composition of macrophage subtypes for each cancer type. (D) Pie chart displays the proportion of various macrophage subtypes present in each cancer type. (E) Heatmap showing the expression levels of EPGM and TPGM in different macrophage subtypes across carcinoma in situ. The heatmap is color‐coded to represent the relative expression levels, with blue indicating low expression and red indicating high expression. (F and G) Boxplots showing the expression level of EPGM (F) and TPGM (G) in different macrophage subtypes. The boxplots display the median (line inside the box), interquartile range (box), and whiskers indicating the range of the data.

We analyzed subsets comprising 51,882 Mac cells and 39,249 Mono cells and classified them into 20 subtypes based on differentially expressed genes. These subtypes were designated using the most significant genes, including C1Q^hi^ Macro, CD16^+^/CD52^+^ Mono, CXCL10^+^ Macro, CXCL9^+^ Macro, FTL^+^ Macro, HES2^+^ Macro, HLA‐DRB1^+^ Macro, HMGB2^+^ Mono/Macro, HSPA^hi^/STAT1^+^ Mono/Macro, IL1B^+^ Mono, IL1B^+^/CD44^+^ Mono, ISG15^+^/ISG20^+^ Mono, LYZ^+^ Mono, MT1X^+^ Macro, MT2A^+^ Macro, S100A4^+^ Mono, S100A^hi^ Mono, S100A^hi^/LST1^+^ Mono, TREM2^+^ Macro, and TREM2^+^/LIPA^+^ Macro (Figure 3A,B). The subset distribution varied across tumors. HLA‐DRB1^+^ Macro (29.2%) and ISG15^+^/ISG20^+^ Mono (27.5%) were prevalent in the THCA group. BRCA showed a dominant CXCL10^+^ macrophage level (57.7%). CRC featured TREM2^+^ macrophages (38.9%) and IL1B^+^ monocytes (25.6%). LUAD contained 48% of TREM2^+^ macrophages. HCC, OV, and PRAD were dominated by HES2^+^ (83.7%), FTL^+^ (79.7%), and MT1X^+^ macrophages (94.1%), respectively (Figure 3C,D).

Next, we assessed the expression of TPGM and EPGM in various tumor types. Notably, in BRCA, the expression of TPGM and EPGM was upregulated in the CXCL10^+^ Macro, C1Q^hi^ Macro, HSPA^hi^/STAT1^+^ Mono/Macro, CD16^+^/CD52^+^ Mono, FTL^+^ Macro, HLA‐DRB1^+^ Macro, and HMGB2^+^ Mono/Macro groups. In HCC cells, TPGM and EPGM expression levels were elevated in the HMGB2^+^ Mono/Macro, HES2^+^ Macro, S100A^hi^ Mono, LYZ^+^ Mono, and TREM2^+^ Macro groups. LUAD showed increased TPGM and EPGM expression in the MT1X^+^ Macro and TREM2^+^/LIPA^+^ Macro groups. In the OV, TPGM and EPGM expression levels were upregulated in IL1B^+^/CD44^+^ Mono and ISG15^+^/ISG20^+^ Mono cells, with TPGM specifically elevated in IL1B^+^ Mono cells. In THCA, TPGM and EPGM expression increased in MT2A^+^ Macro, S100A4^+^ Mono, and S100A^hi^/LST1^+^ Mono cells. Finally, in HCC cells, TPGM and EPGM expression levels were upregulated in the S100A4^+^ Mono and MT2A^+^ Macro (Figure 3E). Our findings showed increased TPGM and EPGM expression in BRCA macrophage subsets. Quantitative analysis confirmed this trend across subsets. Notably, the HSPAhi/STAT1^+^ Mono/Macro subset showed a significantly higher expression than the other subsets (Figure 3F). Previous BRCA studies found HER2 overexpression and amplification in 15%–25% of cases, linked to worse prognosis with lower disease‐free and overall survival rates [46, 47, 48]. The elevated expression of TPGM and EPGM may be a potential influencing factor contributing to this phenomenon.

### 3.4 Expression of TPGM and EPGM Varies Across Disease Stages and Is Influenced by Treatment

**FIGURE 4:**
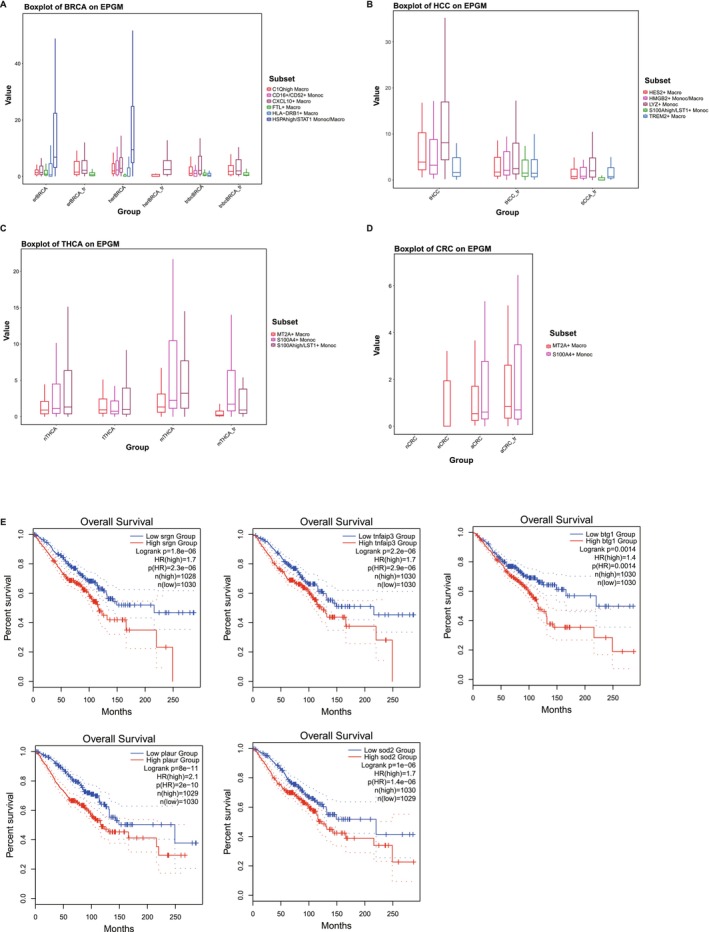
Expression of EPGM varies across tumor stages and is influenced by treatment. (A) Boxplot depicting the expression of EPGM on breast cancer (BRCA). (B) Boxplot depicting the expression of EPGM on Hepatocellular Carcinoma (HCC). (C) Boxplot depicting the expression of EPGM on Thyroid Cancer (THCA). (D) Boxplot depicting the expression of EPGM on Colorectal Cancer (CRC). (E) Kaplan–Meier depicting the variation in overall survival among different genes exhibiting high expression in tumors.

We analyzed TPGM and EPGM expression across tumor stages and treatment effects. In ER/HER single‐mutant BRCA, HSPAhi/STAT1^+^ Mono/Macro vanished post‐treatment, indicating the suppression of therapeutic angiogenesis (Figure 4A, Figure S1A). In HCC, some cell subtypes with initially high TPGM/EPGM expression levels decreased post‐treatment. Notably, S100A^hi^/LST1^+^ Mono emerged post‐treatment but with low TPGM/EPGM, suggesting alternative roles (Figure 4B, Figure S1B). In THCA, S100A4^+^ Mono cells displayed higher TPGM/EPGM in treated/recurrent lymph nodes and distant metastases than in para/localized/advanced tumors. Notably, EPGM expression was significantly increased in S100A^hi^/LST1^+^ Mono cells in treated/recurrent lymph nodes. (Figure 4C, Figure S1C). Additionally, in CRC, S100A4^+^ Mono and MT2A^+^ Macro showed persistently high TPGM and EPGM expression in advanced tumors and after treatment, suggesting novel prognostic and therapeutic avenues for patients with CRC (Figure 4D, Figure S1G).

To explore the potential impact of EPGM on cancer patient prognosis, we conducted a systematic analysis of overall survival using GEPIA2 [49]. Specifically, in three different types of tumors—BRCA, THCA, and LUAD—we assessed the overall survival of several EPGM genes, including *SRGN*, *TNFNIP3, BTG1*, *PLAUR*, and *SOD2*. We found a significant decrease in overall survival among patients with high expression of these genes, strongly suggesting that elevated EPGM expression in tumors may indicate a higher risk of mortality (Figure 4E). Our study offers new insights into the role of EPGM in tumor occurrence, development, and prognosis, providing valuable clues for future research. However, it is important to note that while our analysis suggests an association between these genes and prognosis, further experimental validation and in‐depth investigations are necessary to elucidate the specific molecular mechanisms involved.

## 4 Discussion

Angiogenesis plays a crucial role in tumorigenesis, serving as a prerequisite for tumor growth, metastasis, and lethality. A comprehensive understanding of its mechanism holds immense significance for the diagnosis, treatment, and prognosis evaluation of tumors. Recent research has unveiled remarkable parallels between early embryonic development and tumorigenesis, encompassing phenomena such as cell migration, gene expression patterns, intricate signaling pathways, cellular differentiation dynamics, immune evasion strategies, and other related aspects. However, whether the mechanism underlying tumor angiogenesis mirrors that of embryonic angiogenesis remains inadequately investigated. Macrophages assume a pivotal function in tumor angiogenesis and have also been implicated in embryonic angiogenesis. Previous work has shown that the process of angiogenesis comprises various stages and entails the coordinated action of growth factors, substrate molecules, and diverse cell types, among which TAMs play a critical role [50]. Exploring the parallels between macrophage‐mediated angiogenesis in embryonic development and its function in tumor progression could pave the way for innovative therapeutic strategies targeting angiogenesis in cancer.

In this study, we conducted a comprehensive pan‐cancer analysis of scRNA‐seq obtained from 332 patients, encompassing a total of 804,406 cells across seven different human cancer types. Through this analysis, we identified nine major lineages characterized by canonical cell markers. Subsequently, we performed a detailed examination of subsets containing 51,882 macrophages and 39,249 monocytes, delineating them into 20 distinct subtypes based on their differential gene expression profiles. Our analysis has definitively shown that EPGM is ubiquitously present in tumors, and our findings have further uncovered a strikingly diverse expression pattern of both EPGM and TPGM across these distinct subtypes. Furthermore, the subtypes that exhibit high expression of these two gene modules vary significantly across different tumors. This observation implies that when developing therapeutic strategies such as anti‐angiogenic therapy, it is imperative to consider the distinct characteristics of various cell subtypes. This recognition suggests novel strategies and directions for targeting angiogenesis in the treatment of tumors, potentially leading to more effective and personalized therapeutic approaches.

The assessment of tumor angiogenesis pre‐ and post‐treatment serves as a crucial criterion for evaluating treatment efficacy [51, 52]. Hence, we conducted a comparative analysis of EPGM and TPGM expression levels in various tumor types before and after treatment. We observed that the expression levels of EPGM or TPGM in most cell subtypes were relatively decreased after treatment, potentially indicating a favorable prognostic outcome. For instance, in ER single‐mutant and HER single‐mutant BRCA, we observed that the expression levels of both EPGM and TPGM in the HSPA^hi^/STAT1^+^ Mono/Macro were significantly attenuated after treatment (Figure 4A, Figure S1A). However, both cell populations were absent in TNBC, a subtype of BRCA Clinical evidence suggests that TNBC typically results in poorer clinical outcomes. These findings imply that targeting proangiogenic myeloids in BRCA treatment may yield therapeutic benefits. On the other hand, we noted that there were several cell subtypes, such as S100A4^+^ Mono in THCA, S100A4^+^ Mono, and MT2A^+^ Macro in CRC, which highly expressed EPGM or TPGM, but the expression level of both modules did not decrease after treatment. Therefore, developing potential therapeutic strategies for these tumors could involve effectively controlling the proangiogenic module expression in these cell subtypes. In brief, our study provided insight into understanding the mechanisms driving angiogenesis in associated cancers. Those observations may provide novel perspectives for assessing the effectiveness of anti‐angiogenic therapy and facilitate the further improvement of treatment strategies. By taking into account the heterogeneity of cell subtypes and their response to treatment, we can potentially develop more targeted and effective therapeutic approaches against tumor angiogenesis.

The close relationship between tumorigenesis and developmental biology has been proposed by Pierce in the 1970s [53]. Subsequent investigations revealed a significant number of human embryonic genes being reactivated in cancer cells [54]. As advancements in molecular biology, tumor immunology, developmental biology, and experimental embryology have unfolded, a substantial body of evidence has emerged, affirming the correlation between embryonic development and tumorigenesis. Recent research has highlighted remarkable parallels between early embryonic development and tumorigenesis across various aspects [24]. The findings of our study are poised to offer valuable insights into understanding the intricate mechanisms underpinning aberrant angiogenesis, which is crucial for formulating precise interventions aimed at hindering tumor progression.

## Author Contributions

**Zeshuai Wang:** conceptualization (equal), data curation (equal), formal analysis (equal), methodology (equal), project administration (equal), visualization (equal), writing – original draft (equal), writing – review and editing (equal). **Yiyi Su:** conceptualization (equal), data curation (equal), formal analysis (equal), writing – original draft (equal). **Lisha Zhao:** formal analysis (equal), validation (equal), visualization (equal). **Wei Liu:** formal analysis (supporting), project administration (supporting), visualization (supporting). **Jiaqi Zhang:** data curation (supporting), formal analysis (supporting), visualization (supporting). **Wei Yang:** data curation (supporting), project administration (supporting), visualization (supporting). **Hanjie Li:** investigation (equal), validation (equal). **Mingqian Feng:** conceptualization (equal), formal analysis (equal), project administration (equal), visualization (equal), writing – original draft (equal), writing – review and editing (equal). **Hao Wang:** conceptualization (equal), data curation (lead), project administration (equal), validation (equal), writing – original draft (equal), writing – review and editing (equal). **Zhuo Song:** conceptualization (equal), data curation (equal), formal analysis (equal), project administration (equal), validation (lead), visualization (equal), writing – original draft (equal), writing – review and editing (equal).

## Conflicts of Interest

The authors declare no conflicts of interest.

## Supporting information


**Figure S1.** Expression of TPGM and EPGM varies across tumor stages and is influenced by treatment (A) Boxplot depicting the expression of TPGM on breast cancer (BRCA). (B) Boxplot depicting the expression of TPGM on hepatocellular carcinoma (HCC). (C) Boxplot depicting the expression of TPGM on thyroid cancer (THCA). (D) Boxplot depicting the expression of EPGM and TPGM on ovarian cancer (OV). (E and F) Boxplot depicting the expression of EPGM (E) and TPGM (F) on lung cancer (LUAD). (G) Boxplot depicting the expression of TPGM on colorectal cancer (CRC).

Data S1.

Data S2.

## Data Availability

All scRNA‐seq data are downloaded from public database (Data IDs are: GSE184362, GSE141445, GSE131907, GSE151530, GSE132465, GSE178318, GSE176078, E‐MTAB‐8107, E‐MTAB‐6149, and E‐MTAB‐6653). The curated data are available from the corresponding author on reasonable request.
