# Supplementary material for: Pan‐Cancer Single‐Cell Transcriptomic Analysis Reveals Divergent Expression of Embryonic Proangiogenesis Gene Modules in Tumorigenesis
Source: Cancer Med. 2024 Nov 11;13(21):e70373. doi: 10.1002/cam4.70373 (PMC11551789; doi:10.1002/cam4.70373)
Supplement: Supplementary file 1 — Figure S1. Expression of TPGM and EPGM varies across tumor stages and is influenced by treatment (A) Boxplot depicting the expression of TPGM on breast cancer (BRCA). (B) Boxplot depicting the expression of TPGM on hepatocellular carcinoma (HCC). (C) Boxplot depicting the expression of TPGM on thyroid cancer (THCA). (D) Boxplot depicting the expression of EPGM and TPGM on ovarian cancer (OV). (E and F) Boxplot depicting the expression of EPGM (E) and TPGM (F) on lung cancer (LUAD). (G) Boxplot depicting the expression of TPGM on colorectal cancer (CRC). [file CAM4-13-e70373-s003.pdf]

Fig S1

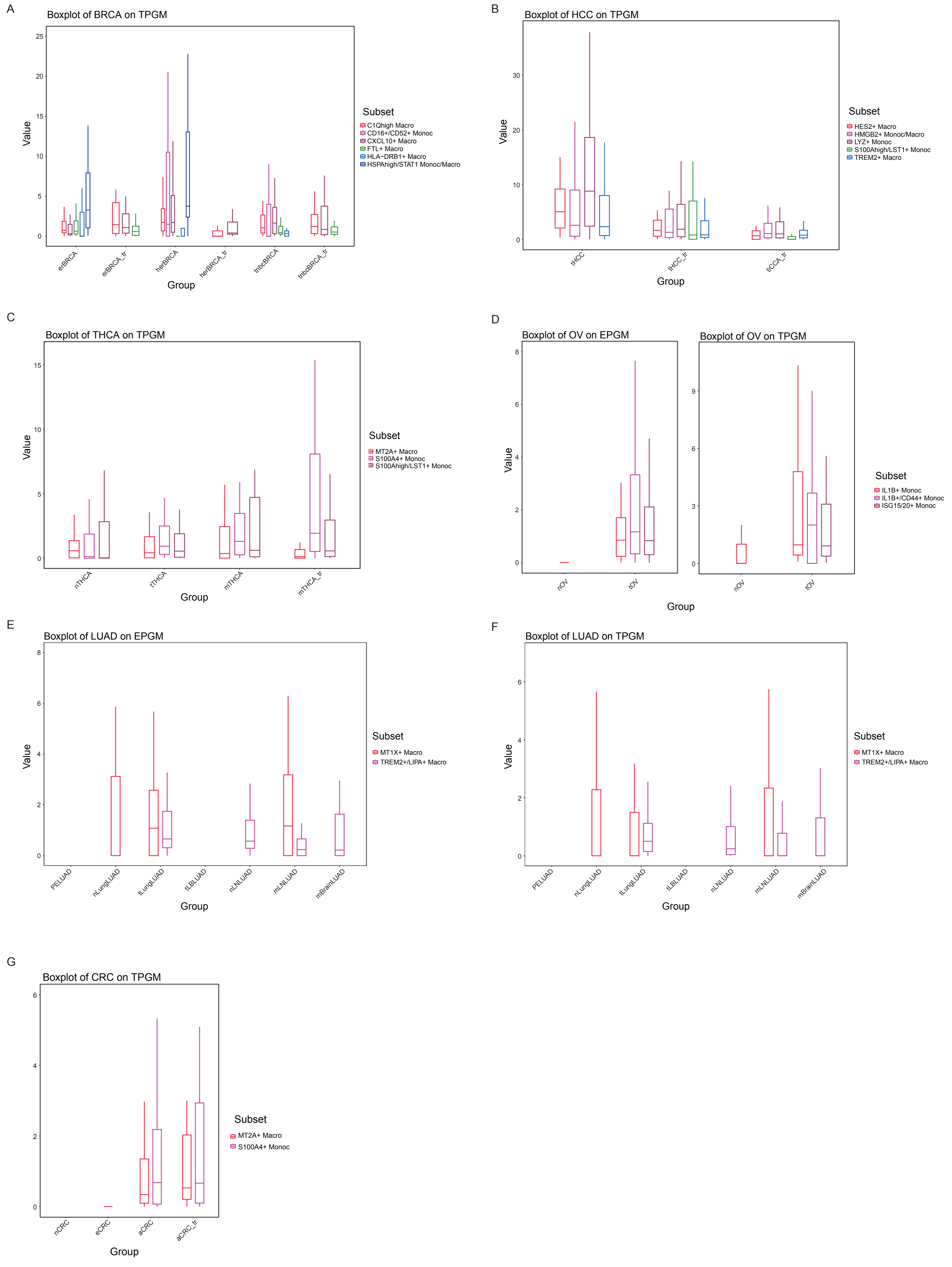
A
Boxplot of BRCA on TPGM
Value
Group
erBRCA
erBRCA_tr
herBRCA
herBRCA_tr
tnbcBRCA
tnbcBRCA_tr
Subset
C1Qhigh Macro
CD16+/CD52+ Monoc
CXCL10+ Macro
FTL+ Macro
HLA−DRB1+ Macro
HSPAhigh/STAT1 Monoc/Macro
B
Boxplot of HCC on TPGM
Value
Group
tHCC
tHCC_tr
tiCCA_tr
Subset
HES2+ Macro
HMGB2+ Monoc/Macro
LYZ+ Monoc
S100Ahigh/LST1+ Monoc
TREM2+ Macro
C
Boxplot of THCA on TPGM
Value
Group
nTHCA
tTHCA
mTHCA
mTHCA_tr
Subset
MT2A+ Macro
S100A4+ Monoc
S100Ahigh/LST1+ Monoc
D
Boxplot of OV on EPGM
Boxplot of OV on TPGM
Value
Group
nOV
tOV
Subset
IL1B+ Monoc
IL1B+/CD44+ Monoc
ISG15/20+ Monoc
E
Boxplot of LUAD on EPGM
Value
Group
PELUAD
nLungLUAD
tLungLUAD
tLBLUAD
nLNLUAD
mLNLUAD
mBrainLUAD
Subset
MT1X+ Macro
TREM2+/LIPA+ Macro
F
Boxplot of LUAD on TPGM
Value
Group
PELUAD
nLungLUAD
tLungLUAD
tLBLUAD
nLNLUAD
mLNLUAD
mBrainLUAD
Subset
MT1X+ Macro
TREM2+/LIPA+ Macro
G
Boxplot of CRC on TPGM
Value
Group
nCRC
eCRC
aCRC
aCRC_tr
Subset
MT2A+ Macro
S100A4+ Monoc

Figure S1: Expression of TPGM and EPGM varies across tumor stages and is influenced by treatment

A. Boxplot depicting the expression of TPGM on breast cancer (BRCA). B. Boxplot depicting the expression of TPGM on Hepatocellular Carcinoma (HCC). C. Boxplot depicting the expression of TPGM on Thyroid Cancer (THCA). D. Boxplot depicting the expression of EPGM and TPGM on Ovarian Cancer (OV). E and F. Boxplot depicting the expression of EPGM(E) and TPGM(F) on Lung cancer (LUAD). G. Boxplot depicting the expression of TPGM on Colorectal Cancer (CRC).
